# “We are in the forgotten corner!” a qualitative study of experiences and challenges among Chinese older women at the onset of acute myocardial infarction

**DOI:** 10.3389/fpubh.2023.1242322

**Published:** 2023-09-22

**Authors:** Huidan Yu, Huafen Liu, Zifen An, Jiali Zhou, Xianmei Meng, Xianwu Luo, Xiaoyang Zhou

**Affiliations:** ^1^School of Nursing, Wuhan University, Wuhan, Hubei, China; ^2^Department of Cardiology, Renmin Hospital of Wuhan University, Wuhan, Hubei, China

**Keywords:** myocardial infarction, gender, experience, challenges, older people, qualitative

## Abstract

**Background:**

Acute myocardial infarction (AMI) is a common and serious cardiovascular disease (CVD) that is one of the leading causes of death among women globally and in China. However, there are sex-associated differences and inequalities in the detection and management of AMI, especially in older people. There is little research demonstrating how challenges and barriers affect older women’s help-seeking behavior and health-related procedures in China.

**Purpose:**

The objective of this study was to explore the experiences of older women with AMI, focusing on their perception, challenges, and coping strategies at the onset of AMI in Wuhan, China.

**Methods:**

This study utilized a qualitative research design approach and conducted semi-structured, in-depth, and audio-recorded interviews with 18 women aged 65–84 years, purposively selected from two tertiary hospitals in Wuhan City from November 2021 to April 2022.

**Results:**

Interpretative Phenomenological Analysis (IPA) was used in this study to analyze the data on 18 participants and three major themes were generated: disease perception disorder, negative coping strategies, and barriers due to social-environmental contexts.

**Conclusion:**

To reduce older women’s delay in seeking help, healthcare professionals should provide public health education that emphasizes sex-related disparities, and age-specific knowledge-attitude aspects to high-risk groups. Policy-based and health administration recommendations, including e-health information support, access to care, and social-environmental factors, should be highlighted to promote women’s health behavior.

## Introduction

Acute myocardial infarction (AMI) is an important cause of death globally, causing over 7 million deaths globally each year (1). The morbidity of AMI in China was lower than in Western countries in the past, but changes in social movement and lifestyle have caused a 7.5% increase in cardiovascular disease prevalence in China (2). Additionally, China will experience overall growth and aging of its adult population in the coming decades. According to the results of the Seventh National Population Census in China in 2020, 13.5% of the population is aged 65 years old or above. By 2030, the population is predicted to reach 1.46 billion, with 16% of Chinese citizens being aged 65 years old or above (3). These risk factors significantly increase the incidence of morbidity and disease burden of MI in China (4–6).

In patients with AMI, symptoms and their interpretation are important early indicators of the need to seek medical care. Older people often have atypical clinical manifestations, diversified symptoms, and higher mortality, which make them prone to be misdiagnosed and underdiagnosed, especially in the female population (7, 8). Studies have shown that women’s risk of dying during the first 2 weeks after a heart attack is double that of men (9, 10), and delay in seeking medical help (>2 h) is more than threefold higher in women aged ≥65 years compared to other adult women (10, 11).

One meta-analysis research used a qualitative thematic synthesis approach on 21 articles to synthesize the experiences of patients with AMI with delays in seeking treatment and found that decision-making in seeking medical help is a complex social and psychological process. This process included interpreting and evaluating symptoms, which were influenced by cognitive, emotional, and cultural aspects (12). The interpretation of symptoms could influence their behavior of seeking help, and the patients who considered their symptoms to be serious, urgent, and caused by cardiac events tended to seek medical treatment earlier (12–14). Female patients, on the other hand, often lacked familiarity with the symptoms associated with AMI and experienced confusion over the symptoms, which were factors that could be a major barrier to seeking care (13–15).

Dreyer et al. conducted a meta-synthesis of 17 articles on patients’ experience after AMI and found that participants had to navigate the emotional reaction, but lacked professional guidance to help them cope with the psychosocial changes in their lives (16). Limited qualitative studies have shown that women adopt more passive coping strategies than men, such as depressive reactions, denial, and minimizing the impact of the disease (17–19). In addition, some research on the medical-seeking decision experiences of patients with AMI did not focus on the female population and few studies have explored the individual nature and inner thoughts of older women in particular (12, 16, 18, 19).

Many older Chinese women prioritize taking care of their partners and children over their own health status, despite having critical chronic diseases. Moreover, the majority of Chinese women undertake the double burden of household duties and work and often interpret symptoms as fatigue from overwork rather than as a painless manifestation of AMI; therefore, they tend to delay seeking treatment, which might cause a pre-hospital delay (10, 20, 21). In addition, studies have pointed out that older women in China tend to seek medical advice from friends and neighbors rather than healthcare professionals or have online consultations (9, 10, 22). Due to China entering an aging society in the year 2000, an increasing number of older people have faced increasing challenges and barriers in the adoption of health care access in the Internet era (23), therefore, psychosocial processes can influence the decision about seeking medical help, and sex-related, age-specific, ethnic, and cultural backgrounds may be significant factors.

Some Chinese researchers studied female patients’ knowledge, attitudes, beliefs, and responses to AMI symptoms, and the limited research used qualitative methods to explore the age-specific female experience of AMI, focusing on the perceptions, challenges, thought processes, and coping strategies used by older women in mainland China. Given these issues and using a sample of Chinese older women with AMI, this study aimed to address the following questions: How did Chinese older women describe their experiences and challenges at the early stages of AMI? What kind of factors and barriers could affect their decision-making process in seeking medical care?

## Methodology

### Design and sample

This study adopted Leventhal’s common sense model (CSM) as the theoretical framework, which illustrated that patients could form a preliminary cognitive representation after they were aware of disease threat and health perception, and formulated corresponding action plans to cope based on cognitive representation (24). It was used to understand the interviewees’ awareness, emotional response, and behavior regarding health information and experience of coping with illness. As little information about the topic is available in China, a qualitative method with a descriptive phenomenological approach was used to explore the experiences, perceptions, and challenges in older women during the early stages of AMI under the background of the information age.

The target population was inpatients in cardiovascular wards from two tertiary hospitals affiliated with a university in the central part of China. Participants were recruited through purposive sampling. The inclusion criteria were: (1) age ≥ 65 years, (2) women with a medical diagnosis of AMI, (3) mentally competent for communication, (4) able to understand and read Mandarin Chinese, and (5) approximately 24–48 h post admission.

### Data collection

A semi-structured interview was held with the participants privately in a single room. The researcher utilized open-ended and reflective questions to probe participants’ experiences that pertained to some of the awareness, thought processes, responses, and coping strategies of women with AMI. The following questions were utilized: What were you doing when you felt uncomfortable? What did you think when the symptoms happened? What factors affected your decision to seek help for AMI symptoms? What challenges and barriers did you experience while going to see a doctor? Could you describe your experience of admission to the hospital? What do you know about acute myocardial infarction?

The sequence of the questions was not the same for each participant, depending on the individual interview process and the answers given by the participants. The interview varied according to the issues emerging from the data as the interviews and analysis progressed.

The interviews took 40–55 min and were audio-recorded with the express permission of the participants. Immediately following each interview, contextual information was recorded, such as the participant’s manner of speaking and non-verbal behavior. The researcher transcribed each audio recording verbatim.

### Data analysis

Interpretative Phenomenological Analysis (IPA) was used in this study to analyze the data (25). IPA focuses on the complexity and process of individual experience and aims to explore how people perceive, understand, and construct health and disease, which is one of the common research methods in phenomenology. It was suitable for understanding individual experiences from the perspective of specific groups in a specific context.

The analysis steps included: (1) Extensively recording open annotated texts, (2) identifying themes from the preliminary annotations, (3) extracting information and naming topics, (4) identifying inter-theme associations, (5) writing characteristic reports and starting the next case analysis, and (6) identifying thematic patterns in individual cases.

After repeated and careful examination of the participants’ transcripts by two researchers, major categories and key themes were developed and defined. The interviews were conducted until unique and new themes were no longer identified (26). Each theme was coded and each code was compared with others to identify differences and similarities in all the data. Saturation occurred with the 16th interview, in which all levels of coding were complete and no new theme emerged. Two other interviews were done to ensure saturation.

### Quality control

Guba and Lincoln proposed that the trustworthiness and rigor of qualitative research can be measured by four criteria: credibility, dependability, confirmability, and transferability (27). To ensure credibility, one researcher in this study was a nurse with many years of experience in the cardiology department. The researcher obtained the trust of female patients while providing care to them every day; thus, interviews were formed under the relationship of trust, and the credibility and accuracy of the data were greatly improved. In the interview process, the researchers recorded the interview content completely and transcribed it as soon as possible after the interview to form a preliminary description. The transcript would be returned to the female patient for verification and preservation. To enhance dependability, NVivo 11 was adopted to manage the data to facilitate the audit trail. To ensure confirmability, the researchers repeatedly checked and rechecked statements with the interviewees, for example, “Do you mean...” was used to avoid the subjective intervention of researchers during the interview. In this way, the interview data could present as closely as possible the opinions and reasonings of the interviewees. Detailed study procedures, methods, and main characteristics of participants were provided to enhance the transferability of findings across contexts. The interview outline was used to elicit the feelings and experiences of female participants during the early stages of AMI, and the description content was further explored to obtain the essential description.

### Ethical consideration

Ethical approval (WHU-2021-YF0010) was acquired by the institutional review board of Wuhan University and its affiliated teaching hospitals before the study. Following site approval from the Department of Cardiology, the researcher invited eligible female patients to participate in the study on an individual and voluntary basis and explained the objective and methods of the study. The patients had the right to withdraw from the study at any time. The audio recording did not begin until the patient’s oral and written consent was obtained. The interview was held in a private room to maintain privacy. Confidentiality was assured by anonymously coding all data by numbers. Tapes and transcripts were kept in locked files, which only two researchers could access.

## Results

### Description of the participants

A total of 18 participants were interviewed, with ages ranging from 65–84 years, with a mean of 70.22 years (SD 4.98). Three participants (16.67%) had received a tertiary diploma and above, six (33.33%) had completed high school education, four (22.22%) had completed middle school education, and five (27.78%) had obtained only a primary school education (see Table 1). All of them were retired, but two were still working full-time in the family business. Among them, two participants were smokers, one drank alcohol every day, and the others had never smoked or drunk alcohol. Of the participants, 4 were widows and lived alone, and 14 lived with their husbands or family members.

**Table 1:** Basic information of interviewees.

Patient number	Age	Educational background	Marital status	Living with	Delay in hours
1	66	Tertiary	Married	Husband and family member	3.4
2	67	High school	Married	Husband	5.6
3	84	Primary school	Widow	Living alone	13.8
4	72	Middle school	Widow	Living alone	7.9
5	65	Tertiary	Married	Husband	14.3
6	68	High school	Married	Husband	23.1
7	69	High school	Married	Husband	18.9
8	73	Primary school	Widow	Living alone	19.6
9	70	Primary school	Married	Husband	21.4
10	68	Middle school	Married	Husband	16.2
11	70	High school	Married	Husband	9.3
12	72	Primary school	Married	Husband	6.7
13	75	Primary school	Married	Husband	15.4
14	65	Bachelor degree	Married	Husband and family member	21.6
15	68	Middle school	Married	Husband	19.9
16	80	Primary school	Widow	Living alone	35.8
17	69	High school	Married	Husband	24.0
18	72	High school	Married	Husband and family member	15.8

Three major themes were generated from the participants’ narration and the data analysis, namely, disease perception disorder, negative coping strategies, and barriers to social-environmental context. Several subthemes were also illustrated with quotes (see Table 2).

**Table 2:** Extracting information and naming themes and sub-themes.

Theme 1 Disease perception disorder	
Sub-themes	Quotes
Failure to recognize the atypical symptoms	*“I went to the drug store at once and bought the antacids when I feel sick, vomited, and sweated; I thought I might take some unsanitary food this morning.”(N. 2)* [*sic*] *“I had toothache for nearly 2 days, I did not want to eat anything, the dental problem is a common health problem in old people… to my surprise, I was told I had AMI rather than the dental problem.”(N. 12)* [*sic*]
Unawareness of cardiac risk	*“I never thought that I would have a heart attack because I am a female and I never smoke.”(N. 1)* [*sic*] *“We often heard about well-known actresses or female singers who died from breast cancer or cervical cancer, but we rarely heard about them dying from cardiac attack.”(N. 6)* [*sic*]
Theme 2 negative coping strategies	
Denial	*“My brother had two experiences of heart attacks before and he thought my symptom is similar to his, but I do not think so, why me? It is impossible for me to have a coronary disease … I could not accept that I had a heart problem.”(N. 9)* [*sic*] *“I feel something was wrong, I had a sensation of something pressing on my chest all day, I decided not to think about it and waited for the symptom disappear.”(N. 10)* [*sic*]
Incorrect self-help behavior	*“I was sure it was indigestion; I had the same symptom 2 weeks ago… stomached, nausea and diaphoresis, I asked my husband to go to the drug store and buy some promoting digestion medicine for me.”(N. 17)* [*sic*]
Ambivalence	*“Although I felt uncomfortable, I did not want to bother others.”(N. 8)* [*sic*] *“I do not know how to solve this problem; I am not sure what happened to me.… I could not let my family drop in a mess.” (N. 13)* [*sic*]
Theme 3 barriers due to social-environmental contexts	
Lacking professional information support	*“I telephoned my friend when I felt dizziness and sweating.… I followed her advice and waited for several hours, but it did not work…, I should see a doctor earlier.” (N. 7)* [*sic*] *“My sister told me she recovered soon after taking this medication; so I went to the drug store with her and bought the same medication, in fact, the medication has no effect on me, I still feel palpitation and weakness.” (N. 16)* [*sic*]
Obstacle to online medical services	*“My daughter told me she often registers and consults for the physician online, but I could not use WeChat or web-based platforms like that… Maybe I would receive treatment early if I could finish the online consultation.” (N. 11)* [*sic*] *“I do not dare to see the doctor without my son or daughter-in-law; I had to rely on their help. I have to wait until they are free to take me to the hospital.”(N. 8)* [*sic*]
Prioritizing family responsibility over seeking help	*“I feel bad yesterday morning, but I did not tell my family member. … Therefore, I told myself I have to tolerate uncomfortably. My family will drop into a mess if my health condition became deteriorated.*” *(N. 18)* [*sic*] *“Why I went to see the doctor so late? … I must take on family and household responsibilities, and I do not want to be a burden on my family.” (N. 14)* [*sic*]

### Disease perception disorder

#### Failure to recognize atypical symptoms

From their own recollection, the majority of the participants had experienced atypical symptoms, such as nausea, vomiting, weakness, fatigue, sweating, dizziness, dyspnea, jaw pain, back pain, and chest discomfort, but they contributed these signs to gastrointestinal or psychosomatic problems and did not believe these symptoms were associated with a heart attack. One informant (N. 2) said: *“I went to the drug store at once and bought the antacids when I feel sick, vomited, and sweated, I thought I might take some unsanitary food this morning, so I took the medication and went to bed because I felt tired at that time … I thought I would be ok after good rest”*[*sic*]. Many informants stated that they felt discomfort at the onset of their symptoms, perceiving that “something was wrong,” but they did not immediately recognize that discomfort as a heart problem and that they should see a doctor as soon as possible. Another informant, a 72-year-old (N. 12) said*: “I had toothache for nearly 2 days, I did not want to eat anything, the dental problem is a common health problem in old people, my daughter visited me by chance at that day and decided to take me to see a dentist, to my surprise, I was told I had AMI rather than the dental problem”* [*sic*].

#### Unawareness of cardiac risk

In all, 83.3% of participants said that they never thought they would have a heart attack, even among healthcare workers. One informant (N. 1) said: “I was a retired nurse in a stomatology hospital. I took my health condition seriously; I did physical checkups annually especially the examination of breast cancer and uterine myoma…you know, women were at high risk of these diseases. But I never thought that I would have a heart attack because I am a female and I never smoke, I have a good habit of diet and I do physical exercise every day, my figure is fitness, so I believed I must be tired when I was short of breath and had heart palpitation due to heavy workload”[*sic*]. During data collection and analysis, the study found that many participants lacked awareness about the impact of coronary heart disease (CHD) on women and held stereotypical views that heart attack patients should be overweight, smokers, and middle-aged age men. One participant (N. 6) told the author that she could not trust the physician in the cardiac care unit (CCU) because young physicians might make the wrong diagnosis and that everyone knew AMI was a male disease. She said: “You know, we often heard about well-known actresses or female singers who died from breast cancer or cervical cancer, but we rarely heard about them dying from cardiac attack, which is a male-dominated disease” [*sic*].

### Negative coping strategies

#### Denial

Denial perhaps is one of the most commonly used psychological mechanisms for coping with serious threats and with strong emotional stress. Of the 18 women who took part in the interviews, half of them stated that at the onset of their chest discomfort, they initially denied that symptoms might signify anything serious or could relate to heart problems. *One informant (N. 10) said, “I feel something was wrong, I had a sensation of something pressing on my chest all day, I guess it was going to rain, I decided not to think about it and waited for the symptom disappear, so I had a hot bath and drank some milk and went to bed”* [*sic*]. *Another informant (N. 9) said: “my brother asked me to go to the hospital, he has had two experiences of heart attacks before and he thought my symptom is similar to his, but I do not think so, why me? It is impossible for me to have a coronary disease … I could not accept that I had a heart problem, I will be well after good rest, I think …”* [*sic*]. Some informants told the interviewer that they had not believed what the physician said to them at all at first and that they thought the physician took their symptoms to be more serious than they were. However, they finally believed and accepted that they had had a heart attack after several blood examinations and EKG results.

When cardiac symptoms persisted, a few informants recognized something was wrong, or that their symptoms might be related to the heart, but they used denial and avoidance as coping strategies. Many of the women reported that they often used coping strategies, such as wishing or praying that symptoms would go away, trying to relax, pretending nothing was wrong, and trying not to think about the symptoms when they first noticed their symptoms. *One 65-year-old woman (N. 5) said: “I knew it is possible for a woman has heart disease, but being a woman, it is more likely to get gynecological problems than have a heart attack. The number of females got heart attacks is few; I thought I will not become one of them, my mother does not have it, and my family members do not have this kind of disease. Therefore, I told myself I was not that sick when I feel chest discomfort, I attributed the discomfort to tiredness and stress these days. The symptoms would go after good relaxation”* [*sic*].

#### Incorrect self-help behavior

In the present study, 72.2% of patients stated that they decided not to immediately seek professional medical care, preferring self-medication instead at the onset of their symptoms. One interviewee (N. 17) said: *“I was sure it was indigestion; I had the same symptom 2 weeks ago… stomached, nausea and diaphoresis, I asked my husband to go to the drug store and buy some promoting digestion medicine for me*” [*sic*]. There were sex-related differences in the symptoms of AMI, and the symptoms of older female patients were atypical, including fatigue, back pain, loss of appetite, palpitation, and dizziness, and self-treatment was diverse, ineffective, and delayed the patients from seeking medical help. Two interviewees had body massages to relieve back pain and fatigue; three interviewees chose to drink red tea to solve ingestion problems and fatigue, and others took painkillers and drank milk to treat toothache and stomachache.

#### Ambivalence

Though most of the interviewees felt uncomfortable and doubtful when the prodromal symptoms appeared, they were hesitant to see a doctor and tried to delay medical treatment as much as possible to avoid bothering other family members’ work and routines. *One informant (N. 8) said: “Although I felt uncomfortable, I did not want to bother others, I live alone, my daughter is busy and has two children to take care of, I would feel sorry for my daughter if she takes care of me rather than her children, but I had a strong bad feeling, I should see a doctor”* [*sic*]. Female patients expressed in the interview that they were more concerned with family responsibilities and obligations than the importance of their own health and tried to keep a balance between the undertaker of family affairs and the role of the care receiver. *One 75-year-old woman (N. 13) said: “What can I do? I do not know how to solve this problem; I am not sure what happened to me. My husband is 81 years old, he will be anxious if he knew I was uncomfortable. We only have one son who worked in another city that is far from here over 500 kilometers. I could not let my family drop in a mess, maybe it is a false alarm”* [*sic*].

### Barriers due to social-environmental contexts

#### Lacking professional information support

Although it is convenient for the participants who live in a city to see a doctor because there are many clinics and community hospitals available in China, Chinese people, especially retired women, traditionally often share health information and seek advice from family members, and friends rather than health professionals when they have an ailment. One informant with type 2 diabetes (N. 7) said: *“I telephoned my friend when I felt dizziness and sweating, she is also a diabetes patient, and she told me I had hypoglycemia and suggest that I should take orange juice or eat a few biscuits. I followed her advice and waited for several hours, but it did not work…actually, I should see a doctor earlier, there is a community hospital nearby my house, I go there by walk for no more than 10 min”* [*sic*]. Another interviewee (N. 16) stated*: “My sister told me she recovered soon after taking this kind of medication; her symptom was similar to me, so I went to the drug store with her and bought the same medication, but, in fact, the medication has not effect on me at all, I still feel palpitation and weakness”* [*sic*].

#### Obstacle to online medical service

Most large teaching hospitals in China have developed online registration booking systems to provide convenience and quick medical service for clients. However, many older people face difficulties using electronic payment and online registration services. One informant (N. 11) stated: *“My daughter told me she often registers and consults for the physician online, it is save-time and efficient for medical advice and treatment, but I could not use WeChat or web-based platforms like that… this is hard for us older people. Maybe I would receive treatment early if I could finish the online consultation”* [*sic*]. Another 72-year-old woman (N. 4) said*: “I went to the hospital and found I just sit around and do nothing, the nurse asked me to show the electronic registration information, but I did not know how to perform it on my cell phone, so I sit there and waited for the nurse to help me, but many older people need this kind of help, I had to wait and do nothing”* [*sic*]. Some participants complained that hospitals’ online registration system is so complex that they could not go to the hospital by themselves. One older lady (N. 8) said: *“I do not dare to see the doctor without my son or daughter-in-law, I had to rely on their help, but they are very busy. I have to wait until they are free to take me to the hospital”* [*sic*].

#### Prioritizing family responsibility over seeking help

During the interview, most of the informants (77.8%) expressed a strong notion that their work and family role function was influenced by their health status. One 72-year-old woman (N. 18) said*: “Actually, I feel bad yesterday morning, but I did not tell my family member. I have to take care of my husband, he had a stroke 3 years ago, and I also have to do the cooking for my granddaughter, she is studying in middle school and she has lunch at my house every day, so I told myself I have to tolerate uncomfortably. My son and daughter-in-law are very busy; my family will drop into a mess if my health condition became deteriorated”* [*sic*]. Most of the participants expressed that they were not willing to bother others with their health problems because of their own and others’ role expectations of family responsibilities. A 65-year-old woman (N. 14) said: *“Why I went to see the doctor so late? You see, this is the end of the year, and my husband is a part-time faculty in college, he has a lot of work to do, a lot of annual summaries and reports to finish. I could not disturb him at that time. My little son is going to have a master entry examination this month which is a very significant moment for him; I must take on family and household responsibilities, and I do not want to be a burden on my family*” [*sic*].

## Discussion

### Older women’s delay in seeking medical help for AMI

According to the results of the Global Burden of Disease Study 2019 (28), cardiovascular disease is the leading cause of death among women, accounting for 35% of all deaths among women. Approximately 25% of women died of CHD in the US, and 50% of women were more likely than men to be initially misdiagnosed at the onset of heart attack symptoms (29). The hospitalization rate of female patients with AMI in China has increased nearly four-fold from 2001 to 2011 (30), and the incidence of in-hospital cardiac death was significantly higher in female patients than in male counterparts (15, 31). The total ratio of major adverse cardiac events (MACE) and mortality during hospital was higher in the older female group than in other adult female groups (10, 15, 30). Cardiovascular disease in women is called a silent killer, it remains underestimated, under-recognized, and under-diagnosed globally and especially in China (2). Reducing pre-hospital delay, early diagnosis, and optimal treatment are widely recognized as critical steps in decreasing mortality from AMI. In this study, the time between the onset of AMI symptoms and admission to the hospital ranged from 3.4 h to 35.8 h, and the mean delay time was 16.26 ± 7.94 h. The result of the study indicated that delays in seeking care in older female participants were related to not recognizing atypical symptoms, denying initial symptoms, holding the incorrect perception of the disease, and inappropriate self-treatment, which is consistent with other studies (14, 32, 33).

### Age- and sex-related education programs focus on the knowledge-attitude-behavior aspect

Based on the report from the Lancet Women and Cardiovascular Disease Commission, some significant under-recognized risks, including depression, perceived stress, lower economic status, health literacy, and sociocultural role disproportionately influenced by the sex of the patient, contribute to CVD in women (2). Women’s experiences surrounding the onset of AMI symptoms and the decision-making process related to seeking medical help were influenced by several factors, such as psychosocial context and cognitive-emotional response to the symptoms (2, 12, 18, 19, 32). Research has indicated that most women are more likely to believe they are more vulnerable to breast cancer than cardiac disease, whereas the truth is that there is a 31% likelihood that a woman may experience cardiac disease in her life while only a 2.8% likelihood of her developing cancer (34). In the interviews conducted in the present study, 66.7% of participants reported a difference between the symptoms they experienced and the symptoms expected. The perception that heart disease is a male-dominant health problem is reinforced by the media in China, which often choose middle-aged men as target people in advertisements to arouse public concern about cardiac disease. In addition, as a result of the influence of traditional Chinese culture, breast cancer or gynecological health problems are often thought of as a greater threat to women than heart attacks, and some health professionals and patients still tend to underestimate and under-recognize the cardiac risk in women (32). The result of this research demonstrated that knowledge gaps and cognitive differences in detection, prevention, and access to care for women still exist nowadays (2, 7), which is consistent with the results of other studies (16–19).

Social-environmental factors being involved in the decision about seeking treatment as well as cultural differences might be an aspect influencing health behavior. Very few women are full-time housewives in China, and while most Chinese women have the same workload as men, they spend an extra 2–3 h performing household chores, which is approximately 43.9% more time than men spend in these activities, and 85.3% of women have reported excessive workload and working stress (9, 20, 21). In Chinese traditional culture, as an old famous saying states that “women uphold half of the sky.” Chinese women work just as hard as men and have been smoking more than before, with many women interpreting fatigue, weakness, loss of appetite, and palpitation as a result of overworking rather than as painless atypical manifestations at the onset of AMI, which causes a delay in the decision-making time for seeking medical care (32). Some participants said in the interview that they had to take on heavier household and working responsibilities after retirement, meet others’ role expectations, and neglect their own ailments or tolerate the discomfort when they were at the onset of symptoms. Sutantri et al. (19) found that their sample of women avoided telling relatives about their physical discomfort out of concern for others and for fear of not being allowed to undertake what they perceived as their normal household roles.

Most participants in the interview expressed that they lacked information on cardiovascular disease in the female population and the majority of them obtained information from friends, relatives, advertisements, and television. Chinese media often choose men for representations of typical crushing chest pain in TV or advertisements, which could mislead awareness and perception of heart disease among the female population. Media plays a significant role in highlighting factors and behaviors that affect health outcomes. Research suggested that women still have worse outcomes than men after heart attacks and the highest rates of mortality shift from men to women especially those with lower social-demographic indexes. In addition, women were more likely to be susceptible to health resource disparities, access to care, and continuity of treatment than men (5, 35, 36).

In the present study, only one participant directly called a health professional and called up emergency medical service (EMS), whereas the other participants tended to seek advice from family members or friends. Zhang et al. (9) undertook a study of 803 patients with AMI who were admitted to 21 hospitals in China and discovered that although guidelines strongly recommend the use of EMS by patients with AMI, only 39.5% of patients called up the EMS at the onset of symptoms and only 13.1% of female patients utilized EMS, which is one of the reasons of the pre-hospital delay in female patients. Yin et al. (37) used face-to-face individual interviews in Guangdong Province to explore patient-level and system-level barriers associated with delay in AMI treatment and found patient-level barriers included poor knowledge in recognizing AMI symptoms and not calling EMS when symptoms occurred. Future intervention strategies are imperative to strengthen publicity and health education, promote MI patients’ health behavior by using EMS timely, and increase EMS capacity to improve health outcomes.

### Help the older population adopt mobile health technology

Adequate health literacy is related to detecting and managing disease risk and contributes to good health behavior and utilization of healthcare services. With the advent of the information age, e-health literacy is becoming more imperative. E-health literacy refers to the ability of individuals to utilize emerging information and communication technology to improve health outcomes and access to healthcare services. The survey by Li et al. (22) showed that the qualified rate of e-health literacy among 1,201 older adults was 11.1% and that inadequate e-health literacy contributed to poor health outcomes and low utilization of healthcare services.

Almost every participant in the interview process of the present study mentioned the difficulties and challenges they encountered in completing medical service procedures online, such as appointment booking, service guidance, and report checking. These measures were considered very convenient, time-saving, and efficient in the younger patients group, but for the older population, it was a big challenge. With mobile technology, most tertiary hospitals in China are trying to improve smart systems of medical service, registration, and treatment to decrease people gathering, reduce waiting time, and improve the medical service experience. Online registration has become the mainstream way to make appointment bookings including the use of hospitals’ mobile apps, WeChat official accounts, and other online booking systems, but it can be a barrier for older people who might not be as familiar with smartphones as younger people (23). In the study conducted by Zhu et al. (38), the authors suggested that the development of the Internet had brought great convenience to seek medical treatment for general clients, but not for older people, those with more health issues, and the most frequent users of health services who do not have access to the Internet. The “inconvenience” brought to the older group by the appointment network in hospitals is a typical manifestation of the “digital divide” or “technology discrimination” (23).

Poor health literacy is a common public health problem in developing countries, but it also happens in developed countries. An investigation conducted in Germany demonstrated lower health literacy and less use of health care services in women than in men with cardiovascular disease (39). Another study indicated that the patient’s sex was a predictor for poor health literacy at an older age and that it is necessary to ensure women improve self-care, modify risk behavior, and adhere to treatment (40, 41). Thus, it is imperative to promote culturally tailored education and standardized health information practices to detect and manage cardiovascular disease among older women.

Given the increasing difficulties and inconveniences of access to mobile healthcare services that some older people face, policymakers and health professionals should develop measures to optimize the medical care process for the older group. First, hospital administration should reserve a certain number of offline registration appointments and provide offline health services for older people, especially those who seek medical treatment alone, and open telephone appointments, develop devices and hospital access methods that are deemed more friendly to the older population, which is conducive to solving the difficulties and building on age-friendly society (37). Moreover, the experts in health software services should consider age-related needs and regulate the digital products for the older group, such as adding voice guidelines and decreasing manual complexity to improve user experience and convenience. Third, volunteers should strengthen community-based education for mobile health technology for older residents. This is a strong recommendation to encourage young people to provide bottom-up intergenerational digital feedback to older people to narrow the digital gap faced by them.

## Conclusion

In conclusion, this study explored the experiences, perceptions, and barriers among the older female population when having an AMI in China. The findings of this study indicated that older women had to meet the challenges of knowledge-attitude-behavior aspects. Women were more likely to experience health inequality than men due to social, cultural, and economic factors. By exploring the patient-level barriers and unmet needs from the perspectives of older women, this study provided richer and more insightful evidence for the future development of interventions to reduce the burden of cardiovascular disease in women. Efforts should be focused on helping health professionals promote educational programs and culturally tailored communication mechanisms that can address age-specific, gender-related strategies that target older women with cardiovascular disease risk factors to reduce care-seeking delay, improve public e-health literacy and behavior, and promote women’s health quality.

### Limitations

This study has a few limitations. First, the interviews were carried out in two hospitals in one big city in China, which possibly does not reflect cultural diversity. Furthermore, due to the characteristics of qualitative research, the limited sample size may not be representative of all older women with AMI in China. Further research needs to utilize mixed research methods to examine the direct and indirect effect of sex-related, psychosocial factors on older women with MI and to develop and evaluate the intervention strategies on knowledge, perception, and attitude on the behavior of seeking treatment.

## Data availability statement

The datasets presented in this article are not readily available because they may contain information that could compromise research participants’ privacy and consent. Requests to access the datasets should be directed to XL, luoxw187@126.com.

## Ethics statement

The studies involving human participants were reviewed and approved by Wuhan University (WHU-2021-YF0010). The participants provided their written informed consent to participate in this study.

## Author contributions

HY: conceptualization, methodology, validation, formal analysis, and writing – original draft. HL: conceptualization, methodology, investigation, and formal analysis. ZA: formal analysis and validation. JZ: methodology, investigation, and formal analysis. XM: conceptualization, resources, and validation. XL: methodology and conceptualization. XZ: methodology and writing – review and editing. All authors contributed to the article and approved the submitted version.
